# Facts *Versus* Teaching

**Published:** 1899-05

**Authors:** S. B. Palmer

**Affiliations:** Syracuse, N. Y.


### FACTS *VERSUS* TEACHING.

BY DR. S. B. PALMER, SYRACUSE, N. Y.

In the regents' dental examination representing the New York State Dental Society, the following question, in various forms, has been repeated relating to chemistry and metallurgy: In what order should metals be fused in making dental alloy to avoid volatilization? In comparing answers for several examinations the average would be about seven, to fuse the tin first, and add silver, copper, gold, etc., against ten, to commence with the high-fusing metals and descend according to the fusibility of the constituents. Figures show that more than fifty per cent. of the answers are wrong. I do not make this criticism against teachers or text-books, nor in any way to locate the source from which the error comes. It is most surprising that facts have been so long overlooked, since the principle has been promulgated many years.

The following quotation from "Plastics and Plastic Fillings," by J. Foster Flagg, page 62, edition of 1881, clearly defines the process, which is an embodiment of practical facts: "For the making of alloy, the hessian, or sand crucible, is used. In this is first fused a very liberal portion of borax, sufficient in amount to fill the crucible at least one-third full of the molten salt. This is intended for a flux. Any ordinary coke- or coal-fire is all that is required for the 'melt;' but it is, of course, more systematically, and perhaps more readily, done at the usual dental, or smelting forge-fire. Having perfectly fused the borax in it, *the tin is melted*

*first*, requiring but a low temperature, and after it is melted the granulated silver is added.

"It is remarkable, when the high fusing point of silver is considered, with what facility this metal is taken up by the molten tin. These two metals are thoroughly stirred together with an iron rod or clay pipe-stem of small size and suitable length, and when completely incorporated the copper—small pieces of wire—is added. Thus, like the silver, notwithstanding its first fusing-point of almost 2000° F., it is soon melted, and may be equally homogeneously mixed. Lastly the gold is added, melted, and all are thoroughly stirred together with the iron rod or pipe-stem. When perfectly melted and mixed, the fused mass should be quickly poured into a broad, open, flat, shallow matrix, made of iron or soap-stone; this favors prompt cooling, and thus secures the greatest uniformity of distribution to the components."

Platinum has not been included in the above quotation. Still it is used, and, to be accurate in regard to fusing this metal in tin, I made three experiments; the results may be relied upon without fail, and would be of value to any one in fusing platinum in tin over the old method.

A piece of pure platinum was taken of 32 gauge and one inch wide. Strips were cut from the end about one-eighth of an inch wide. The pieces were then passed through the rolls until they were about four inches long, which was as thin as the mill would roll accurately. The strips were cut three inches long. Borax was placed in a small sand crucible which might hold one and a half ounces of tin. It was placed over a Bunsen burner, with the intention of fusing the borax. The heat was not sufficient to even form a glazing. The salts rolled up in a white puff, and was removed. The tin was melted and the platinum dipped in, being held at one end by tweezers. On removal of the portion immersed in the molten tin, the edges were jaggy and holes appeared in places upon the surface. Thus, by dipping, the metal was dissolved to the stub in twelve minutes.

The fact that the surface was not equally tinned was proof that flux was needed. Another crucible was used, with borax for the flux. The crucible was taken from the coals and carried to the laboratory; the platinum was dipped into the molten salts and tin, and quickly withdrawn. All that had entered the tin was missing; this was repeated, with the same results. The third dip dissolved the platinum to the point held by the tweezers. The time required was less than a minute. Not being satisfied with the first experiment, it was repeated, the object being to find the time required to fuse platinum in tin at a heat barely sufficient for its fusion. The tin was melted over a Bunsen burner without being covered. The platinum strip was dipped into hydrochloric acid, and a drop or two of the acid let fall upon the tin surface, which at once freed it from oxygen. The strip was introduced and came out perfectly tinned. The thin bit of metal was pressed into the tin up to the pliers, being withdrawn occasionally. In just five minutes the platinum had been taken up by the tin. I have been particular in giving the time and conditions. Should the experiment be tried by some one using a thicker piece of metal, and not taking pains to free the platinum from oil or oxide that might be impacted by the rolls, or to remove the oxide of tin from the surface, as was the case with the first experiment, the record might be pronounced a failure. Fortunately such low heat is not required in making alloy. This fact is plainly demonstrated, that no more heat is required for making dental alloy than will liquefy borax for the flux; this includes platinum.

This closes the arguments relating to making alloy. I trust the reader will kindly receive some object-lessons in connection with the statement already made. The principle upon which tin fuses the high-melting metal is the same as that of mercury in the setting of amalgam. Mercury is a metal in fusion in ordinary temperatures; it corresponds to the tin in the heated crucible. It fuses the cuttings of alloy and adds another metal to the mass. Another illustration makes clear the effect of washing or not washing amalgam. In our first experiment the oxide of tin retarded the fusion or setting to twelve minutes. The washing by the acid lowered the time to five minutes. The conditions are analogous to mixing amalgam with or without washing.

